# Secondary prevention of myocardial infarction in people with dementia: a multi-jurisdictional retrospective cohort study in Asia, Europe and the USA

**DOI:** 10.1093/ageing/afag220

**Published:** 2026-08-07

**Authors:** Jenni Ilomäki, George S Q Tan, Xiwen Simon Qin, Anna-Maija Tolppanen, Julian Lin, Tian Tian Ma, Miyuki Hsing-Chun Hsieh, Stella Jung-Hyun Kim, Clara Marquina, Edward Chia-Cheng Lai, Li Wei, Stephen J Wood, Ian C K Wong, Gang Fang, Sirpa Hartikainen, Izabela Annis, Kui-Kai Lau, J Simon Bell

**Affiliations:** Centre for Medicine Use and Safety, Monash University, 381 Royal Parade, Parkville, Victoria 3052, Australia; Centre for Medicine Use and Safety, Monash University, 381 Royal Parade, Parkville, Victoria 3052, Australia; Centre for Medicine Use and Safety, Monash University, 381 Royal Parade, Parkville, Victoria 3052, Australia; Department of Pharmacology and Pharmacy, The University of Hong Kong, Hong Kong Special Administrative Region, China; Laboratory of Data Discovery for Health (D24H), Hong Kong Science Park, Hong Kong Special Administrative Region, China; School of Pharmacy, University of Eastern Finland, Kuopio, Finland; Finland Pure-Fountain Ltd, Hammaslahti, Finland; Department of Pharmacy, Shenzhen Second People’s Hospital, Shenzhen, Guangdong, China; School of Pharmacy, Institute of Clinical Pharmacy and Pharmaceutical Sciences, College of Medicine, National Cheng Kung University, Tainan, Taiwan; Population Health Data Centre, National Cheng Kung University, Tainan, Taiwan; UCL School of Pharmacy, University College London, London, UK; Centre for Medicine Use and Safety, Monash University, 381 Royal Parade, Parkville, Victoria 3052, Australia; Centre for Medicine Use and Safety, Monash University, 381 Royal Parade, Parkville, Victoria 3052, Australia; Population Health Data Centre, National Cheng Kung University, Tainan, Taiwan; UCL School of Pharmacy, University College London, London, UK; UCL School of Pharmacy, University College London, London, UK; Centre for Medicine Use and Safety, Monash University, 381 Royal Parade, Parkville, Victoria 3052, Australia; Department of Pharmacology and Pharmacy, The University of Hong Kong, Hong Kong Special Administrative Region, China; Laboratory of Data Discovery for Health (D24H), Hong Kong Science Park, Hong Kong Special Administrative Region, China; Aston Centre of Excellence in Pharmacotherapy Optimisation and Pharmacoepidemiology Research School of Pharmacy, Aston University, Birmingham, UK; Eshelman School of Pharmacy, The University of North Carolina at Chapel Hill, Chapel Hill, NC, USA; School of Pharmacy, University of Eastern Finland, Kuopio, Finland; Eshelman School of Pharmacy, The University of North Carolina at Chapel Hill, Chapel Hill, NC, USA; Department of Medicine, University of Hong Kong, Hong Kong Special Administrative Region, China; Centre for Medicine Use and Safety, Monash University, 381 Royal Parade, Parkville, Victoria 3052, Australia

**Keywords:** dementia, comparative effectiveness, cohort study, myocardial infarction, secondary prevention, older people

## Abstract

**Background:**

Statins, renin–angiotensin system inhibitors (RASIs) and beta-blockers are guideline-recommended cardiovascular medications post-myocardial infarction (MI) for secondary prevention, but evidence for people with dementia is scarce.

**Objective:**

To evaluate the association between post-MI cardiovascular medication use and the risk of cardiovascular outcomes and mortality, focusing on people with dementia.

**Design:**

Large retrospective cohort study using data from Australia, Finland, Taiwan, the UK and the USA.

**Subjects:**

People with and without dementia.

**Methods:**

Seven exposure groups were assigned using one or combinations of the three guideline-recommended medication classes—(i) statin, RASI and beta-blocker, (ii) statin and beta-blocker, (iii) statin and RASI, (iv) RASI and beta-blocker, (v) statin only, (vi) beta-blocker only and (vii) RASI only, based on medication records during a 60-day landmark period post-MI. Recurrent MI, major adverse cardiovascular event (MACE) and all-cause mortality were evaluated as outcomes. Jurisdiction-specific results were pooled together using meta-analyses.

**Results:**

A total of 28 122 people with dementia and 260 360 people without dementia were included. Among people with dementia, using any single or two medications carried a similar risk of recurrent MI and MACE as using all three guideline-recommended medications, except that using RASI and a beta-blocker without a statin was associated with a lower risk of recurrent MI (HR 0.85; 95% CI, 0.75–0.96). Using a statin and RASI without beta-blockers resulted in similar all-cause mortality to using all three (HR 1.16; 95% CI, 0.97–1.39), while all the remaining single- or dual-medication regimens were associated with higher risks of all-cause mortality.

**Conclusions:**

For people with dementia, using one or two guideline-recommended medications appeared sufficient to protect against recurrent MI and MACE. Beta-blockers may not provide additional survival benefits over statins and RASIs.

## Key Points

- Do people with dementia benefit from the same cardiovascular medications post-MI as people without dementia?
- Retrospective cohort study with data from five jurisdictions, focusing on people with dementia.
- Same protection against recurrent MI and MACE when using one or two of these medications as taking all three.
- Using statins and RASIs, without beta-blockers, associated with similar risk of all-cause mortality.

## Introduction

Dementia affects 55 million people and is responsible for more than 2 million deaths annually [1, 2]. These deaths are partly linked to high rates of cardiovascular disease (CVD). A Taiwanese survey reported that hypertension (53% vs. 50%) and CVD (19% vs. 3.3%) were more prevalent among people with dementia compared to the general population [3]. Finns with Alzheimer’s disease (AD) have a 16% higher risk of coronary heart disease [4] and a 36% higher risk of death after an acute CVD event than people without AD [5]. People with dementia in Spain and Germany also have higher odds of stroke [6, 7].

Management of CVD in people with dementia remains suboptimal [8–10]. Despite preventing all-cause mortality in older adults [11, 12], Australians with dementia are less likely to receive and more likely to discontinue statins than similar-aged people without dementia [13]. Similarly, a Finnish study found that statin use declines after AD diagnosis [14]. This may reflect actual or perceived differences in treatment benefits and risks in people with dementia. However, people with dementia are excluded from or under-represented in major clinical trials, and so evidence specific to people with dementia is scarce [15]. Observational studies using routinely collected healthcare data can supplement evidence for this vulnerable population.

Current clinical guidelines recommend statins, beta-blockers, renin–angiotensin system inhibitors (RASIs) and antiplatelets after an acute coronary event such as myocardial infarction (MI) [16–18]. However, there are no recommendations for the use of these CVD medications post-MI in people with dementia. While the use of these medications for secondary prevention of CVD post-MI has been shown to reduce all-cause mortality in frail older people, the benefit-to-risk ratio of these medications in people with dementia is still debated. A meta-analysis including >130 000 individuals with dementia reported insufficient evidence to support beta-blocker use post-MI [19].

We evaluated the association between guideline-recommended post-MI CVD medication use and the risk of recurrent MI, major adverse cardiovascular event (MACE) and all-cause death in individuals with and without dementia. This multi-database study included parallel analyses of data from Australia, Finland, Taiwan, the UK and the USA.

## Methods

### Data sources

We analysed electronic health records and administrative claims data from Australia, Finland, Taiwan, the UK and the USA using a common study protocol, as part of Four Continents for Dementia research programme of the Neurological and Mental Health Global Epidemiology Network [20]. Data sources and ethics approvals across the five jurisdictions are described in Supplementary Appendix S1.

### Cohorts

The studied cohorts included people (Australia and the UK: aged ≥ 30, Finland: all ages, Taiwan: aged ≥ 18, the USA: aged ≥ 65) discharged from the hospital with a diagnosis of MI between July 2013 and June 2017 (Finland: 2005–2011, the USA: January 2008–December 2010). The index discharge date was defined as the discharge date of the first MI hospitalisation. The first MI hospitalisation was defined as the first inpatient record with a recorded principal or secondary diagnosis of MI (ICD-10 codes: I21–I23, ICD-9 code: 410.×1) during the study period. We used a 5-year lookback period (1 year in the USA) to determine the incidence of MI, and to minimise the inclusion of people with a prevalent history of acute MI. This is important given that the risk of recurrent MI following 1 year post-MI is high [21], and the subsequent risk of CVD outcomes following recurrent MI increases [22]. In the US cohort, we only included people who were enrolled in Medicare for one full year prior to the index discharge date. People were also excluded if they had a record of dialysis, valvular heart disease or rheumatic diseases prior to or at the index discharge date.

People with dementia were defined as having at least one recorded principal or secondary diagnosis of dementia (ICD-10 codes: F00–03, G30, U79.1) or having at least one record of prescribing or dispensing of antidementia medications (ATC codes with N06D) prior to or at the end of the landmark period (Supplementary Appendix S2). During the study period, the Social Insurance Institute of Finland only granted reimbursement for antidementia medications for people meeting specific diagnostic criteria that were in line with the NINCDS-ADRDA and DSM-IV [23]. People without dementia were defined as those without a recorded diagnosis of dementia, or without a prescribing or dispensing record of an antidementia medication prior to or at the end of the landmark period. Specifically in the UK, each person with dementia was matched to up to four people without dementia by age and sex to derive the analytical cohorts.

### Exposure

The exposure of interest was the use of guideline-recommended CVD medications for secondary prevention post-MI, which included statins, RASIs and beta-blockers. Antiplatelets were not included in the analyses, since aspirin (or acetylsalicylic acid), being the main antiplatelet prescribed post-MI, is available over the counter without a prescription. We identified prescribed or dispensed guideline-recommended medications (using ATC codes and local drug codes, see Supplementary Appendix S3) during a 60-day landmark period following the first MI hospitalisation. The 60-day landmark period starts from the index discharge date (Supplementary Appendix S2). Seven treatment groups were assigned using one or combinations of the three medication classes—(i) statin, RASI and beta-blocker, (ii) statin and beta-blocker, (iii) statin and RASI, (iv) RASI and beta-blocker, (v) statin only, (vi) beta-blocker only and (vii) RASI only. People not prescribed or dispensed any CVD medications of interest during the landmark period were excluded.

### Outcomes

People were followed up from the day after the landmark period until the end of the study period, unless any outcome of interest occurred sooner. Outcomes of interest were recurrent MI, MACE and all-cause mortality. Recurrent MI was defined as the first hospitalisation with a principal diagnosis of MI (ICD-10 codes: I21–I23) during follow-up. MACE was defined as the first hospitalisation with a principal diagnosis of MI (ICD-10 codes: I21–I23), heart failure (ICD-10 code: I50), stroke (ICD-10 codes: I60–I64), revascularisation procedures (see Supplementary Appendix S4 for a full list of procedural codes) or CVD-related death. All-cause mortality was identified using the recorded date of death. People who died during the landmark period were excluded.

### Covariates

Demographic characteristics, namely age and sex, were identified at the end of the landmark period. Prior medications were identified using medication prescribed or dispensed within one year prior to the end of the landmark period; concurrent medications were identified during the landmark period. Prior and concurrent medications were defined as having at least two prescribing or dispensing records (see Supplementary Appendix S3). Prior medical diagnoses and procedures were identified using ICD-10 codes in Australia, Finland and Taiwan, ICD-9 codes in the USA and Read codes in the UK (see Supplementary Appendix S4). Frailty was identified using the validated Hospital Frailty Risk Score (HFRS) [24]. Comorbidities and the HFRS were identified and calculated using recorded diagnoses within 5 years prior to or at the end of the landmark period. These covariates are likely associated with the use of guideline-recommended CVD medications post-MI and cardiovascular outcomes and/or mortality, and were included in similar observational studies [25–27].

### Statistical analyses

Baseline characteristics of the study cohorts were described across the five jurisdictions stratified by dementia status. Adjustments for potential confounding factors were performed using the stabilised inverse probability of treatment weighting (IPTW) method [28]. We included demographic characteristics, comorbidities, as well as prior and concurrent medications, in the multinomial logistic regression models to estimate the propensity scores for all seven treatment groups for each individual. The stabilised weights were also truncated at the 1st and 99th percentiles to handle extreme weights [28]. When estimating the risk of recurrent MI and MACE, all-cause death was considered as a competing risk; we used weighted Fine–Gray subdistribution hazard models to estimate hazard ratios (HRs) with corresponding 95% confidence intervals (CIs). To examine the association of CVD medications with all-cause mortality, weighted Cox proportional hazards models were used. The triple combination of statin, RASI and beta-blocker was considered as the reference treatment group for all analyses. Effect estimates were computed for people with and without dementia separately. Additionally, we computed E-values for each HR and corresponding 95% CI, to estimate the minimum strength of unmeasured confounding that would be needed to explain away the observed effect estimates [29].

Meta-analyses were performed to pool results from the five jurisdictions. A random-effects model was selected to conduct meta-analyses using the DerSimonian and Laird method [30], since we expected inherent heterogeneity across the jurisdictions due to differences in population characteristics, prescribing patterns and healthcare systems. Additionally, we have estimated *I*^2^ and τ^2^ statistics as measures of statistical heterogeneity, and conducted Cochran’s Q test to assess the presence of heterogeneity.

All statistical analyses were performed using SAS® software, version 9.4 and R version 4.2.1.

## Results

### Cohort characteristics

This study comprised 260 360 people receiving at least one of the guideline-recommended CVD medications after their first MI hospitalisation, including 32 924 people in Australia, 4474 people in Finland, 56 202 people in Taiwan, 1856 people in the UK and 164 904 in the USA (Table 1). Supplementary Appendix S5 illustrates the flow of cohort attrition across the different jurisdictions. In total, 10.8% (*n* = 28 122) of the study population had a diagnosis of dementia, ranging from 4.3% in Australia to 21.2% in the UK. In Finland, the individuals with dementia comprised 40.7% of the population, given the matched nature of the cohort. The mean age among people with dementia ranged from 73.8 years in Taiwan to 80.9 years in Finland. In Australia and the USA, 76.3% and 81.9% of the cohort were 75 years or older, respectively. The mean age of those without dementia ranged from 63.6 years in Taiwan to 80.8 years in Finland. In Australia, 36.0% of the cohort were 75 years or older, while 55.5% were 75 years or older in the USA.

**Table 1:** Baseline characteristics of study population (people with and without dementia) by jurisdiction.

Characteristics	Australia		Finland		Taiwan		United Kingdom		United States	
	(*n* = 32 924)		(*n* = 4 474)		(*n* = 56 202)		(*n* = 1 856)		(*n* = 164 904)	
	Dementia	No dementia	Dementia	No dementia	Dementia	No dementia	Dementia	No dementia	Dementia	No dementia
	(*n* = 1 414)	(*n* = 31 510)	(*n* = 1 819)	(*n* = 2 655)	(*n* = 6 009)	(*n* = 50 193)	(*n* = 394)	(*n* = 1 462)	(*n* = 18 486)	(*n* = 146 418)
*n* (%)	1 414 (4.3)	31 510 (95.7)	1 819 (40.7)	2 655 (59.3)	6 009 (10.7)	50 193 (89.3)	394 (21.2)	1 462 (78.8)	18 486 (11.2)	146 418 (88.8)
**Post-MI CVD medications (exposure of interest)**										
RASI	884 (62.5)	23 519 (74.6)	1 126 (61.9)	1 999 (75.3)	4 055 (67.5)	34 385 (68.5)	253 (64.2)	1 083 (74.1)	12 358 (66.9)	102 831 (70.2)
Beta-blockers	893 (63.2)	21 799 (69.2)	1 603 (88.1)	2 434 (91.7)	4 014 (66.8)	36 741 (73.2)	288 (73.1)	1 044 (71.4)	15 801 (85.5)	130 086 (88.8)
Statins	1 044 (73.8)	27 872 (88.5)	1 064 (58.5)	1 995 (75.1)	4 193 (69.8)	39 328 (78.4)	309 (78.4)	1 180 (80.7)	13 361 (72.3)	115 458 (78.9)
**Female sex**	722 (51.1)	10 703 (34.0)	1 113 (61.2)	1 571 (59.2)	2 223 (37.0)	11 431 (22.8)	194 (49.2)	712 (48.7)	12 063 (65.3)	76 548 (52.3)
**Age (years)** ^**a**^										
Age (mean, SD)	–	–	80.9 (6.2)	80.8 (5.9)	73.8 (12.2)	63.6 (13.9)	78.2 (11.0)	77.5 (10.6)	–	–
30–64	171 (12.1)	12 341 (39.2)	19 (1.0)	23 (0.9)	–	–	–	–	–	–
65–74	164 (11.6)	7 796 (24.7)	232 (12.8)	354 (13.3)	–	–	–	–	3 334 (18.0)	65 259 (44.6)
75–84	497 (35.1)	7 262 (23.0)	1 072 (58.9)	1 550 (58.4)	–	–	–	–	7 936 (42.9)	56 608 (38.7)
85+	582 (41.2)	4 111 (13.0)	496 (27.3)	728 (27.4)	–	–	–	–	7 216 (39.0)	24 551 (16.8)
**Comorbidities**										
Unstable angina	116 (8.2)	2 259 (7.2)	106 (5.8)	280 (10.5)	–	–	–	–	560 (3.0)	4 714 (3.2)
Other angina pectoris	85 (6.0)	1 872 (5.9)	252 (13.9)	419 (15.8)	–	–	–	–	1 050 (5.7)	7 998 (5.5)
Hypertension	799 (56.5)	14 748 (46.8)	682 (37.5)	1 091 (41.1)	1 419 (23.6)	8 582 (17.1)	255 (64.7)	1 042 (71.3)	–	–
Heart failure	492 (34.8)	6 190 (19.6)	513 (28.2)	725 (27.3)	447 (7.4)	2 310 (4.6)	86 (21.8)	260 (17.8)	5 242 (28.4)	26 638 (18.2)
Atrial fibrillation	387 (27.4)	6 251 (19.8)	356 (19.6)	565 (21.3)	196 (3.3)	952 (1.9)	64 (16.2)	279 (19.1)	2 252 (12.2)	12 938 (8.8)
Stroke	100 (7.1)	946 (3.0)	168 (9.2)	208 (7.8)	1 004 (16.7)	3 075 (6.1)	92 (23.3)	190 (13.0)	2 492 (13.5)	6 912 (4.7)
Peripheral artery disease	86 (6.1)	1 827 (5.8)	197 (10.8)	433 (16.3)	272 (4.5)	851 (1.7)	–	–	4 170 (22.6)	23 521 (16.1)
Major bleeding	185 (13.1)	2 421 (7.7)	81 (4.5)	104 (3.9)	135 (2.2)	651 (1.3)	90 (22.8)	349 (23.9)	–	–
Chronic kidney diseases	599 (42.4)	7 352 (23.3)	72 (4.0)	124 (4.7)	692 (11.5)	3 642 (7.3)	166 (42.1)	531 (36.3)	–	–
Cancer	182 (12.9)	3 708 (11.8)	487 (26.8)	791 (29.8)	428 (7.1)	2 215 (4.4)	56 (14.2)	216 (14.8)	1 752 (9.5)	16 790 (11.5)
COPD	192 (13.6)	2 792 (8.9)	146 (8.0)	223 (8.4)	788 (13.1)	3 618 (7.2)	47 (11.9)	207 (14.2)	4 810 (26.0)	31 852 (21.8)
Diabetes	526 (37.2)	9 198 (29.2)	376 (20.7)	455 (17.1)	796 (13.2)	4 910 (9.8)	132 (33.5)	480 (32.8)	–	–
**Procedures**										
PCI	192 (13.6)	13 680 (43.4)	154 (8.5)	677 (25.5)	2 768 (46.1)	26 883 (53.6)	3 (0.8)	23 (1.6)	488 (2.6)	5 864 (4.0)
CABG	31 (2.2)	2 852 (9.1)	136 (7.5)	286 (10.8)	180 (3.0)	1 791 (3.6)	2 (0.5)	5 (0.3)	53 (0.3)	704 (0.5)
**Frailty**										
HFRS (mean, SD)	6.6 (6.3)	2.3 (3.5)	8.4 (14.7)	1.7 (3.5)	18.5 (17.7)	8.6 (10.9)	8.9 (6.9)^b^	5.0 (6.1)^b^	–^c^	–^c^
**Prior medications**										
Antiplatelets	977 (69.1)	23 281 (73.9)	446 (24.5)	997 (37.6)	2 980 (49.6)	15 207 (30.3)	357 (90.6)	1 215 (83.1)	4 143 (22.4)	27 224 (18.6)
Anticoagulants	217 (15.3)	4 197 (13.3)	335 (18.4)	574 (21.6)	207 (3.4)	1 001 (2.0)	37 (9.4)	214 (14.6)	1 717 (9.3)	12 619 (8.6)
Calcium channel blockers	451 (31.9)	8 620 (27.4)	508 (27.9)	960 (36.2)	2 140 (35.6)	11 726 (23.4)	126 (32.0)	562 (38.4)	6 143 (33.2)	45 437 (31.0)
**Concurrent medications**										
Antiplatelets	905 (64.0)	23 522 (74.6)	667 (36.7)	1 473 (55.5)	5 648 (94.0)	47 782 (95.2)	348 (88.3)	1 180 (80.7)	10 194 (55.1)	95 844 (65.5)
Glucose-lowering medications	333 (23.6)	6 226 (19.8)	484 (26.6)	649 (24.4)	2 486 (41.4)	17 545 (35.0)	57 (14.5)	203 (13.9)	4 036 (21.8)	33,944 (23.2)
Anticoagulants	180 (12.7)	4 129 (13.1)	440 (24.2)	893 (33.6)	324 (5.4)	2 339 (4.7)	22 (5.6)	176 (12.0)	1 934 (10.5)	19 499 (13.3)
Antipsychotics	432 (30.6)	639 (2.0)	726 (39.9)	336 (12.7)	731 (12.2)	2 293 (4.6)	25 (6.4)	22 (1.5)	–	–
Digoxin	72 (5.1)	881 (2.8)	190 (10.4)	279 (10.5)	203 (3.4)	1 541 (3.1)	1 (0.25)	1 (0.07)	–	–
Calcium channel blockers	274 (19.4)	5 985 (19.0)	436 (24.0)	912 (34.4)	1 131 (18.8)	7 005 (14.0)	80 (20.3)	320 (21.9)	4 903 (26.5)	36 896 (25.2)
Loop and thiazide diuretics	549 (38.8)	7 818 (24.8)	1 291 (71.0)	1 899 (71.5)	2 140 (35.6)	12 317 (24.5)	147 (37.3)	547 (37.4)	–	–
Potassium sparing diuretics	93 (6.6)	2 279 (7.2)	222 (12.2)	434 (16.3)	946 (15.7)	6 237 (12.4)	13 (3.3)	74 (5.1)	1 310 (7.1)	12 477 (8.5)
Nitrates	522 (36.9)	14 403 (45.7)	1 413 (77.7)	2 238 (84.3)	–	–	–	–	–	–

Abbreviations: CABG: coronary artery bypass graft, COPD: chronic obstructive pulmonary disease, HFRS: Hospital Frailty Risk Score, MI: myocardial infarction, PCI: percutaneous coronary intervention, RASI: renin–angiotensin system inhibitor.

^a^In Australia and the USA, the age was reported in age-groups rather than the specific age of the patient.

^b^HFRS were presented as median and interquartile range in UK.

^c^HFRS were not calculated for the US cohort.

Hypertension was the most prevalent comorbidity in all of the countries, ranging from 64.7% in the UK to 23.6% in Taiwan in people with dementia, and from 71.3% in the UK to 17.1% in Taiwan in people without dementia (Table 1). The proportion of previous stroke among people with dementia was the highest in the UK (23.3%) and the lowest in Australia (7.1%), while among people without dementia, the UK had the highest prevalence of stroke (13.0%) and Australia the lowest (3.0%). Taiwan had the highest frailty scores for the whole included population (18.5 for people with dementia and 8.6 for people without dementia) and Australia the lowest (6.3 for people with dementia and 3.5 for people without dementia).

Statin was the most commonly dispensed or prescribed post-MI CVD medication in people with and without dementia in Australia (73.8% and 88.5%), Taiwan (69.8% and 78.4%) and the UK (78.4% and 80.7%) (Table 1). Beta-blocker use had the highest prevalence in people with and without dementia in Finland (88.1% and 91.7%) and the USA (85.5% and 88.8%). RASI use was the least prevalent overall, however, it was the second most prevalent CVD medication in Finland (61.9%) and Taiwan (67.5%) among people with dementia and in Australia (74.6%), Finland (75.3%) and the UK (74.1%) among people without dementia.

**Figure 1:**
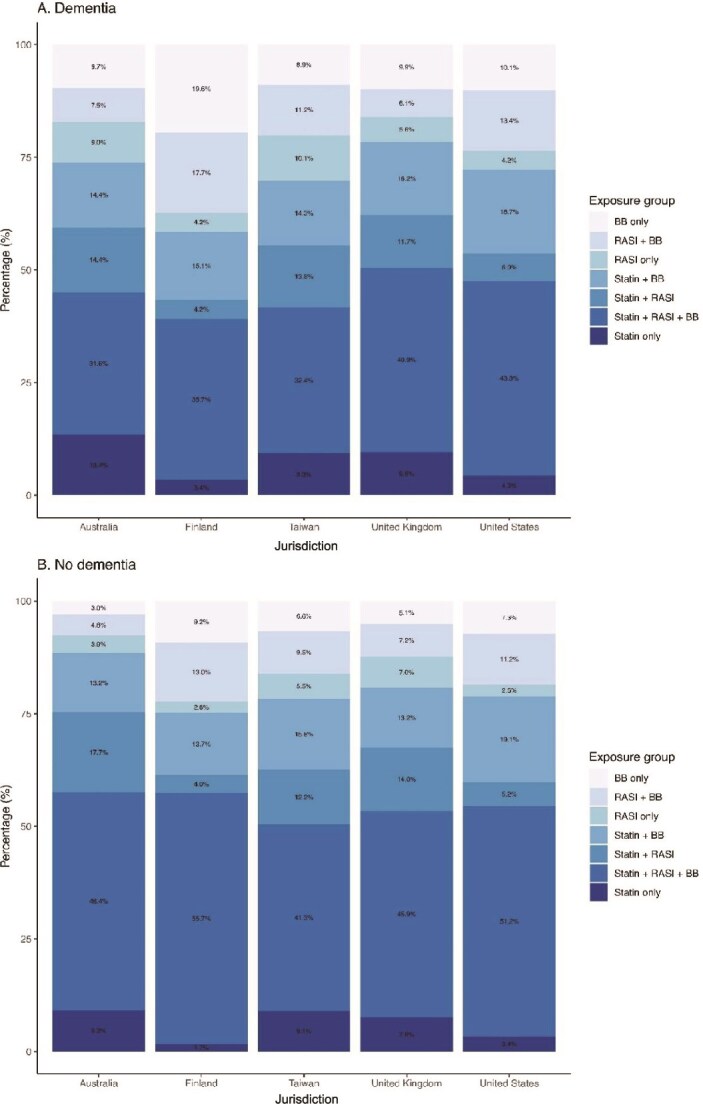
Distribution of treatment groups by jurisdictions. Abbreviations: BB, beta-blocker; RASI, renin–angiotensin system inhibitor.

The most common treatment regimen across all five jurisdictions in both people with and without dementia was all three medications (Figure 1). In people with dementia, the prevalence of using all three medications ranged from 31.6% in Australia to 43.3% in the USA. Comparatively, the prevalence of using all three medications was empirically higher in people without dementia, ranging from 41.3% in Taiwan to 55.7% in Finland. The balance of baseline characteristics between comparison groups was assessed post-weighting. For example, among the Australian cohorts, most baseline characteristics (>90%) were balanced (absolute standardised difference ≤ 0.25) (Supplementary Appendix S6).

### Cardiovascular outcomes and mortality in people with dementia

**Figure 2:**
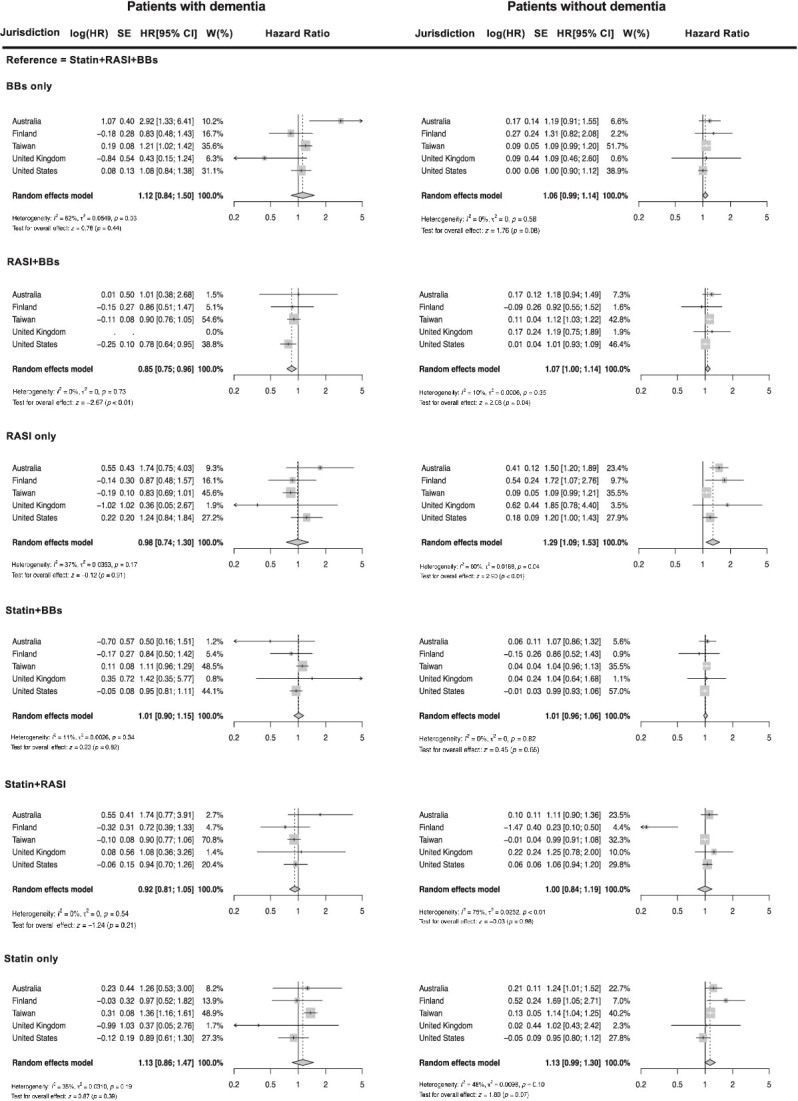
Meta-analyses for the risk of recurrent myocardial infarction. Abbreviations: BB, beta-blocker; CI, confidence interval; HR, hazard ratio; RASI, renin–angiotensin system inhibitor; SE, standard error; W, weight.

In people with dementia, compared to the use of all three medications, none of the dual combinations or single medications showed any significant difference in the risk of recurrent MI (Figure 2). However, the use of a RASI and beta-blockers without statins seemed to significantly reduce the risk of recurrent MI (HR 0.85; 95% CI, 0.75–0.96).

**Figure 3:**
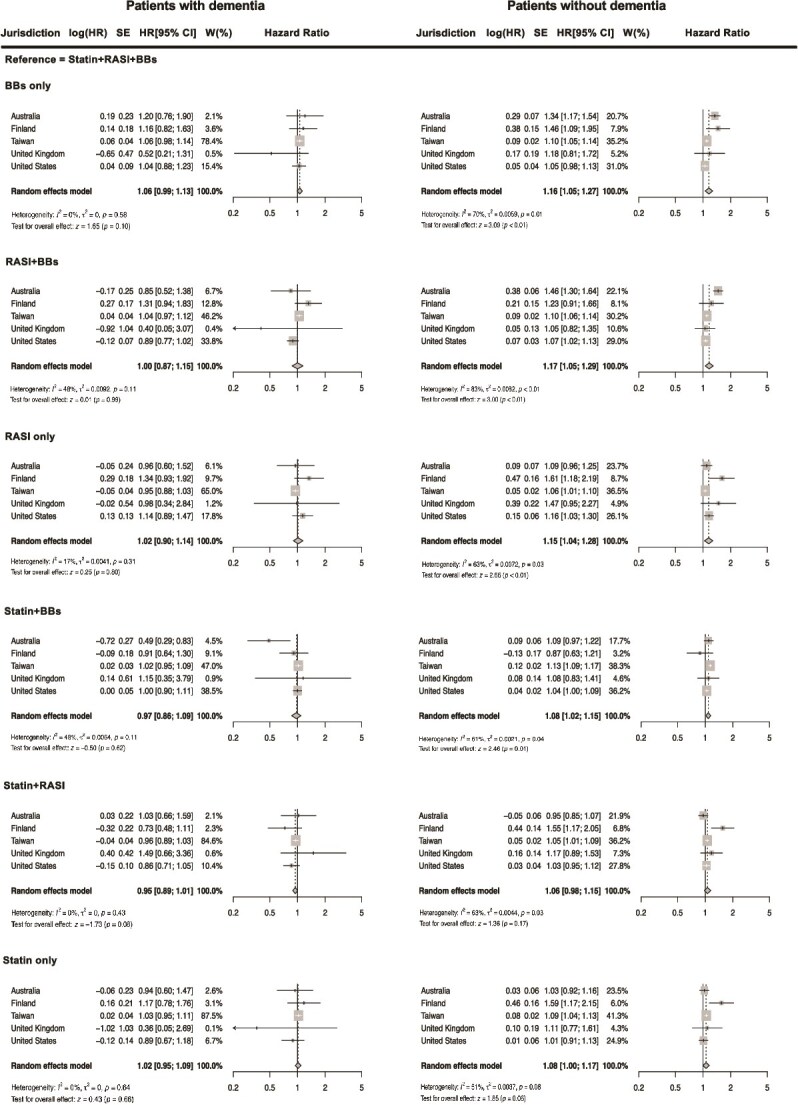
Meta-analyses of the risk of major adverse cardiovascular event*.* Abbreviations: BB beta-blocker; CI confidence interval; HR hazard ratio; RASI renin–angiotensin system inhibitor; SE standard error; W weight.

There were no significant differences in the risk of MACE between the use of all three medications and any of the dual combinations or single medications (Figure 3).

**Figure 4:**
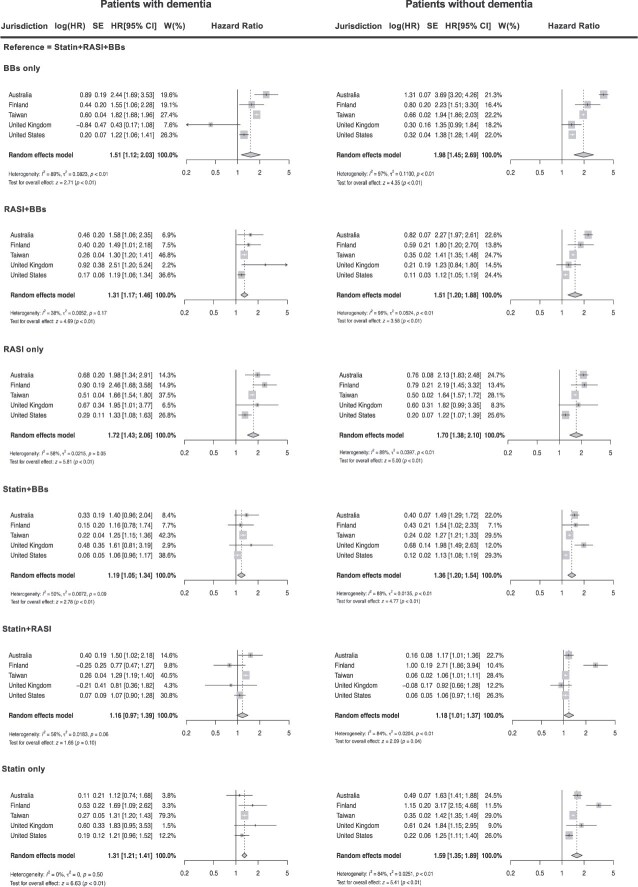
Meta-analyses for the risk of all-cause death. Abbreviations: BB, beta-blocker; CI, confidence interval; HR, hazard ratio; RASI, renin–angiotensin system inhibitor; SE, standard error; W, weight.

In people with dementia, the dual combination of a statin and RASI without a beta-blocker did not significantly increase the risk of all-cause mortality compared to the use of all three medications (HR 1.16; 95% CI, 0.97–1.39; Figure 4). All the remaining dual combination regimens and single medication uses showed a significantly higher risks of all-cause mortality compared to the combination of all three medications.

### Cardiovascular outcomes and mortality in people without dementia

Among people without dementia, compared to the use of all three medications, none of the dual combinations or single medications showed any significant difference in the risk of recurrent MI (Figure 2). However, the risk of recurrent MI in people using RASI only (HR 1.29; 95% CI, 1.09–1.53) was higher compared to people using all three medications.

Compared to the use of all three medications, the combination of a statin and RASI (HR 1.06; 95% CI, 0.98–1.15) and statin-only use (HR 1.08; 95% CI, 1.00–1.17) showed no significant changes in the risk of MACE, while all the remaining combinations showed an increased risk of MACE compared to the use of all three medications (Figure 3).

Among people without dementia, compared to the use of all three medications, all the dual combinations and single medication regimens showed an increased risk of all-cause mortality (Figure 4).

## Discussion

Our findings from more than 25 000 people with dementia and 250 000 people without dementia in the USA, Asia and Europe highlight some differences in the pattern of the association between people with and without dementia. The risk of MACE was no different for any single or two medications compared to using all three medications in people with dementia. However, the risk of MACE was higher when comparing most combinations to all three medications in people without dementia. Furthermore, the risk of all-cause death was not different among people with dementia dispensed statins and a RASI (without a beta-blocker) compared to those dispensed all three medications. However, the risk of all-cause death was higher for statin and RASI use compared to all three medications among people without dementia. Using other combinations of CVD medications was associated with an increased risk of all-cause death when compared to all three medications among people with and without dementia.

Our results substantiate a meta-analysis of two observational studies that reported insufficient evidence to prescribe beta-blockers after MI in people with dementia [19]. One of the included studies enrolled 10 992 nursing home residents admitted to the hospital with an acute MI [31]. The other included study demonstrated that post-MI beta-blocker use reduced all-cause mortality after an MI compared to no beta-blocker use. However, beta-blocker use increased the rate of functional decline among those with moderate-to-severe cognitive impairment (odds ratio 1.34; 95% CI, 1.11–1.61) [31]. We also observed no difference in the risk of recurrent MI or MACE from beta-blocker use in any combination when compared to all three medications among people with dementia. The benefits and risks of using beta-blockers post-MI in people with dementia should be further scrutinised, and tailored clinical recommendations for beta-blocker use should be developed for people with dementia.

Statins reduced all-cause mortality post-MI in both people with and without dementia. However, we observed no difference in MACE when statins were omitted in people with dementia, but an increased risk in people without dementia. A recent meta-analysis reported no evidence for cardiovascular benefits from statins in people with dementia [32]. However, this study also reported no increase in medication-related harms and concluded that the evidence available was insufficient for evaluating the statins’ overall risk-to-benefit profile in people with dementia [32]. Even so, statins may be under-prescribed in people with dementia compared to those without [13], and people with dementia are more likely to stop statin therapy [33]. While these lower prescription rates could be due to an avoidance-of-harm approach by prescribers, our results show that statins are fundamental to, at least, reducing all-cause mortality post-MI in people with dementia.

There was as lower proportion of people with dementia using all three guideline-recommended medications (i.e. statin, RASI and beta-blocker) compared to those without. This is consistent with people with dementia being less likely to be prescribed CVD medications, such as oral anticoagulants [34, 35]. In a study of > 70 000 Finns, people diagnosed with AD were less likely to be prescribed with an oral anticoagulant compared to people without AD [36]. Australians with dementia are also less likely to initiate oral anticoagulants [37]. Further efforts are important to explore the drivers of disparities in CVD medication use between people with and without dementia, and to then devise and implement strategies to address them.

A strength of this study is the large sample size across five jurisdictions with diverse populations, which improves the generalisability of the conclusions. All datasets included individual-level medication records linked to hospital admission or primary care data, which reduced the likelihood of confounding by allowing the control of a wide range of confounders such as comorbidities, co-medications and frailty. The access to high-quality population-based data enabled analysis specific to people with dementia.

Several limitations warrant mentioning. First, although we had access to a large set of covariates and adjusted our analyses for known confounders, residual confounding cannot be ruled out due to unmeasured covariates (e.g. smoking, body mass index, blood pressure, lipid levels). Additionally, some included covariates remained imbalanced after weighting (absolute standardised difference > 0.25) and may contribute to residual confounding. Second, there were inherent differences in population characteristics, prescribing practices and healthcare systems across the jurisdictions, which could introduce heterogeneity in the pooled results. There was substantial or considerable heterogeneity (*I*^2^ > 50%) in many pooled pairwise comparisons. Third, we used records of dementia diagnosis and antidementia medication prescribing or dispensing to identify people with dementia. While this a common approach in observational studies of people with dementia and those evaluating dementia as an outcome [38, 39], there was likely an under-capture of people with dementia, given that dementia diagnoses are often under-recorded and people might not initiate pharmacological treatment. Fourth, we were not able to conduct subgroup analyses by dementia subtype due to the limited number of individuals with non-AD dementia (e.g. vascular dementia). Additionally, the Finnish dementia cohort only included people diagnosed with AD. Last, we only conducted post-hoc subgroup analyses stratified by age and sex in Australia (Supplementary Appendix S7). In people with dementia aged < 75 years in Australia, we observed that there was a significantly higher risk of MACE (HR 3.98; 95% CI, 1.67–9.50) when comparing those who received a beta-blocker only with those who received all three medications. Additionally, there was a higher risk of all-cause mortality (HR 1.64; 95% CI, 1.07–2.52) among males with dementia who received a RASI and statin (but not beta-blocker) when compared to those who received all three medications, but not among females (HR 1.32; 95% CI, 0.80–2.19). Future studies should further investigate effect modification by age and/or sex.

## Conclusion

This is the first large-scale real-world study to investigate the association between guideline-recommended CVD medication use and cardiovascular outcomes following incident MI in people with and without dementia. For people with dementia, using one or two guideline-recommended medications appeared sufficient to protect against MACE, when compared to using all three medications (RASI, beta-blocker and statin). Importantly, our results do not support beta-blocker use post-MI to reduce the risk of recurrent MI, MACE or all-cause mortality. Conversely, RASIs and statins may still be key to reducing post-MI all-cause mortality in people with dementia. However, these findings should not be directly interpreted as evidence supporting reduced therapy intensity. Future studies should explore other clinical benefits and harms of these medications.

## Supplementary Material

aa-25-3261-File006_afag220

## Research Data Transparency and Availability

The raw data of this study are not publicly available as data access was provided by data custodians from the corresponding jurisdictions via secured workspaces under a license agreement.
