# Supplementary material for: Secondary prevention of myocardial infarction in people with dementia: a multi-jurisdictional retrospective cohort study in Asia, Europe and the USA
Source: Age Ageing. 2026 Aug 7;55(8):afag220. doi: 10.1093/ageing/afag220 (PMC13450687; doi:10.1093/ageing/afag220)
Supplement: Supplementary file 1 [file aa-25-3261-file006_afag220.docx]

**Supplementary materials**

**Effectiveness of secondary prevention of myocardial infarction in people with dementia: a multi-jurisdictional retrospective cohort study**

**Table of contents:**

**Appendix 1.** Data sources and ethics approval

**Appendix 2.** Longitudinal cohort study design with landmark period

**Appendix 3.** ATC codes for medications

**Appendix 4.** Diagnosis and procedural codes for baseline comorbidities and procedures

**Appendix 5.** Cohort attrition flowchart

**Appendix 6.** Balance in baseline characteristics in Australian cohorts

**Appendix 7.** Stratified analyses by age and sex in Australia

**References**

**Appendix 1.** Data sources and ethics approval

Australian data were retrieved from the Victorian Admitted Episodes Dataset (VAED). The VAED is a state-wide administrative dataset that contains socio-demographic and clinical information of public and private hospital admissions in Victoria between 2012 and 2018. The VAED was linked to the national Pharmaceutical Benefits Scheme (PBS) to acquire information on prescription medicines that were dispensed through community pharmacies, private hospitals, and public hospitals at hospital discharge. Death information was obtained from the National Death Index. Medical diagnoses were coded according to the International Statistical Classification of Diseases and Related Health Problems, 10^th^ Revision (ICD-10), Australian Modification (ICD-10-AM), and medications were coded according to the World Health Organization’s Anatomical Therapeutic Chemical (ATC) codes and the PBS item codes [1]. Ethical approval for this study was obtained from the Monash University Human Research Ethics Committee (14339) and Australian Institute of Health and Welfare Ethics Committee (EO2018-4-468).

The Finnish MEDALZ (Medication use and Alzheimer’s disease) cohort is a nationwide register-based cohort, which includes 70,719 residents of Finland who were newly diagnosed with AD between 2005 and 2011, and were community-dwelling at the time of AD diagnosis, and a matched comparison person for each person with AD. The matching criteria were same sex, hospital district, and age. The MEDALZ dataset has linkage to the Care Register for Health Care and the Special Reimbursement Register, which provides information on recorded diagnoses in ICD-10 codes from hospital and outpatient care. The Prescription Register of the Social Insurance Institution of Finland provides information on prescription purchases, with medications classified using ATC codes. Data on mortality and socioeconomic status (education, occupational status, and taxable income) were provided by Statistics Finland [2]. According to the Finnish legislation, approval of the ethics committee or informed consent was not required because pseudonymized, routinely collected healthcare data were used and participants were not contacted. Research permit was obtained from the Finnish Social and Health Data Research Permit authority Findata.

The United Kingdom (UK) data were acquired from the Health Improvement Network database (THIN). THIN is a nationwide primary care database that contains anonymised data from over 744 general practices across the UK. It included over 16 million patients, representing about 6% of the UK population. The dataset contained information on demographics, diagnoses, prescriptions, referrals, laboratory tests, immunisations, and the local area deprivation score. Clinical diagnoses, symptoms, investigations and lifestyle information were coded according to the Read codes. Prescription medications were recorded using the British National Formulary codes and generic names, which can be mapped to ATC codes [3]. The study was based on anonymised and unidentifiable THIN data, thus the need for informed consent was waived by the THIN scientific review committee (SRC). This study was reviewed and scientifically approved by THIN SRC (17THIN100).

Taiwanese data were obtained from the National Health Insurance Database (NHID) [4]. This population-based administrative claims dataset covers more than 99% of the Taiwanese citizens and includes records of medical diagnoses, medication prescriptions and clinical procedures from outpatient, emergency department and inpatient settings. Encrypted personal identification number is used to link between data sources. This study has been approved by the Institutional Review Board of National Cheng Kung University Hospital (ID B-ER-108-390).

The United States (US) data comprised of all Medicare beneficiaries between 2008 and 2011. Most citizens and residents of US aged 65 and over are eligible for Medicare. Fee-for-service plans include Part A (hospital coverage), B (outpatient and procedure coverage) and D (prescription medication coverage). Diagnoses in the Medicare data were coded using ICD-9 codes when were mapped to ICD-10 codes. Medication data include the name of the medication, the National Drug Code, quantity reimbursed and date of supply. The project has been reviewed by the Office of Human Research Ethics and was determined to be exempted due to the use of existing, public or deidentified data.

**Appendix 2.** Longitudinal cohort study design with landmark period

**Appendix 3.** ATC codes for medications

| **Drug class** | **ATC codes** |
| --- | --- |
| **Exposure medications** |  |
| Statins | C10AA, C10BA, C10BX |
| Renin-angiotensin system inhibitors (including ACE inhibitors/ARBs) | C09A, C09B, C09C, C09D, C10BX04, C10BX06, C10BX07, C10BX10-C10BX17 |
| Beta-blockers | C07, C09BX02, C09BX04 |
| **Prior medications** |  |
| Antiplatelets | B01AC, C10BX01, C10BX02, C10BX04-C10BX06, C10BX08, C10BX12 |
| Anticoagulants | B01AA, B01AE, B01AF |
| Calcium channel blockers | C08, C09BX01, C10BX03, C10BX07, C10BX09, C10BX11, C10BX14, |
| **Concurrent medications** |  |
| Antiplatelets | B01AC, C10BX01, C10BX02, C10BX04-C10BX06, C10BX08, C10BX12 |
| Glucose-lowering medications | A10A and A10B |
| Anticoagulants | B01AA, B01AE, B01AF |
| Antipsychotics | N05A (excl. N05AN01 lithium) |
| Digoxin | C01AA05 |
| Calcium channel blockers | C08, C09BX01, C10BX03, C10BX07, C10BX09, C10BX11, C10BX14, |
| Loop and thiazide diuretics | C03C (loop diuretics), C03A, C07B, C07D, C09BX03 (thiazides) |
| Potassium-sparing diuretics | C03D, C03E |
| Nitrates | C01DA |

**Appendix 4.** Diagnosis and procedural codes for baseline comorbidities and procedures

| **Comorbidities and procedures** | **ICD-10 codes** |
| --- | --- |
| Coronary artery bypass graft (CABG) | 3530400, 3530401, 3850500, 3530500, 3530501, 3531005, 9020100, 3845619, 3865308, 3849700, 3849701, 3849702, 3850003, 3849704, 3849705, 3849706, 3849707, 3850002, 3850004, 3850302, 3850303, 3850304, 3863700, 3850000, 3850001, 3850300, 3850301, 9022100, 9020101, 9020102, 9020103, Z95.1 |
| Percutaneous coronary intervention (PCI) | 353100, 3531001, 3531002, 3830600, 3830601, 3830602, 3533800, 3534401, 3831200, 3831201, 3831800, 3831801, Z95.5 |
| Unstable angina | I20.0 |
| Other angina pectoris | I20.1, I20.8, I20.9 |
| Hypertension | I10-15 |
| Heart failure | I50 |
| Atrial fibrillation | I48 |
| Stroke | I60-I64 |
| Peripheral artery disease | I70-I73 |
| Major bleeding | D629, I60, I61, I62, I850, K226, K250, K252, K254, K256, K260, K262, K264, K266, K270, K272, K274, K276, K280, K282, K284, K286, K290, K625, K920, K921, K922 |
| Chronic kidney disease | E10.2, E11.2, E12.2, E13.2, E14.2, N00-02, N04-08, N11-12, N14-16, N25-28, T82.4, T86.1, Q60- 63, Z94.0, Z99.2, Z49, N17-19 |
| Diabetes | E10-E14 |
| Cancer | C00-C99 |
| COPD | J44 |

**Appendix 5.** Cohort attrition flowchart

* People who were newly diagnosed with Alzheimer’s disease between 2005-2011 were matched (1:1) with those without Alzheimer’s disease.

** People with dementia were matched (1:4) to those without dementia.

**Appendix 6:** Balance in baseline characteristics in Australian cohorts

| Characteristics | Absolute standardised difference (reference group: RASI + BB + Statin) | | | | | | | | | | | |
| --- | --- | --- | --- | --- | --- | --- | --- | --- | --- | --- | --- | --- |
|  | **People with dementia** | | | | | | **People without dementia** | | | | | |
|  | **RASI + BB** | **RASI + Statin** | **BB + Statin** | **RASI** | **BB** | **Statin** | **RASI + BB** | **RASI + Statin** | **BB + Statin** | **RASI** | **BB** | **Statin** |
| Female | 0.0996 | 0.0281 | 0.0125 | 0.0595 | 0.0024 | 0.0285 | 0.0631 | 0.0226 | 0.0139 | 0.1492 | 0.1747 | 0.0536 |
| Age | | | | | | | | | | | | |
| 30-64 | 0.2024 | 0.0141 | 0.0293 | 0.0718 | ***0.2800*** | 0.1186 | 0.0745 | 0.0224 | 0.0225 | 0.1755 | 0.2057 | 0.0808 |
| 65-74 | 0.1530 | 0.0434 | 0.0642 | 0.1767 | 0.0040 | 0.0193 | 0.0487 | 0.0205 | 0.0106 | 0.0760 | 0.0960 | 0.0182 |
| 75-84 | 0.2333 | 0.0330 | 0.0517 | 0.0068 | 0.0035 | 0.0290 | 0.0393 | 0.0200 | 0.0005 | 0.0712 | 0.0543 | 0.0457 |
| 85+ | 0.0290 | 0.0493 | 0.1133 | 0.1497 | 0.1472 | 0.0855 | 0.1146 | 0.0336 | 0.0453 | 0.2300 | ***0.3006*** | 0.0797 |
| Frailty score (HFRS) | | | | | | | | | | | | |
| <=1 | 0.2471 | 0.0013 | 0.0406 | 0.1815 | 0.1077 | 0.1236 | 0.0872 | 0.0067 | 0.0309 | 0.0734 | ***0.2637*** | 0.0416 |
| >1 and <5 | 0.0007 | 0.0387 | 0.0182 | 0.1623 | 0.0232 | 0.0462 | 0.0201 | 0.0067 | 0.0156 | 0.0237 | 0.0149 | 0.0066 |
| >=5 and <= 15 | 0.1616 | 0.0011 | 0.0013 | 0.0947 | 0.0037 | 0.0412 | 0.0846 | 0.0109 | 0.0546 | 0.1080 | ***0.2630*** | 0.0572 |
| >15 | 0.0353 | 0.0643 | 0.0232 | 0.1147 | 0.1014 | 0.0203 | 0.0288 | 0.0246 | 0.0274 | 0.0649 | 0.1406 | 0.0288 |
| Comorbidities | | | | | | | | | | | | |
| Unstable angina | 0.1500 | 0.0852 | 0.0018 | 0.0972 | 0.0428 | 0.0687 | 0.0061 | 0.0065 | 0.0107 | 0.0139 | 0.0094 | 0.0146 |
| Other angina pectoris | 0.0827 | 0.0543 | 0.0073 | 0.0276 | 0.0087 | 0.0118 | 0.0083 | 0.0011 | 0.0099 | 0.0344 | 0.0309 | 0.0445 |
| Ischaemic stroke | 0.0029 | 0.0347 | 0.0022 | 0.0068 | 0.1793 | 0.0251 | 0.0171 | 0.0043 | 0.0015 | 0.0084 | 0.0549 | 0.0078 |
| Haemorrhagic stroke | 0.0105 | 0.0209 | 0.0465 | 0.0886 | 0.0269 | 0.0184 | 0.0031 | 0.0051 | 0.0078 | 0.0315 | 0.0203 | 0.0114 |
| Heart failure | 0.1428 | 0.0114 | 0.0715 | 0.0022 | 0.0148 | 0.0345 | 0.1036 | 0.0191 | 0.0205 | 0.0933 | 0.1801 | 0.0260 |
| Hypertension | 0.0523 | 0.1102 | 0.0069 | 0.1116 | 0.0846 | 0.0233 | 0.1268 | 0.0076 | 0.0214 | 0.1468 | 0.0696 | 0.0089 |
| Atrial fibrillation | 0.0109 | 0.0370 | 0.0063 | 0.0004 | 0.0345 | 0.0729 | 0.0719 | 0.0084 | 0.0307 | 0.1026 | 0.2113 | 0.0515 |
| Major bleeding | 0.0707 | 0.0365 | 0.0226 | 0.0756 | 0.0877 | 0.0024 | 0.0455 | 0.0030 | 0.0125 | 0.0046 | 0.0598 | 0.0528 |
| Peripheral artery disease | 0.0333 | 0.0033 | 0.0863 | 0.0259 | 0.1123 | 0.0143 | 0.0437 | 0.0086 | 0.0060 | 0.0195 | 0.0722 | 0.0224 |
| Diabetes | 0.0654 | 0.0641 | 0.0332 | 0.0173 | 0.0834 | 0.0464 | 0.0682 | 0.0236 | 0.0200 | 0.0267 | 0.0011 | 0.0385 |
| COPD | 0.0214 | 0.0071 | 0.0363 | 0.0759 | 0.0114 | 0.0119 | 0.0745 | 0.0323 | 0.0256 | 0.1188 | 0.1159 | 0.0675 |
| Cancer | 0.0204 | 0.0365 | 0.1040 | 0.0250 | 0.0377 | 0.0576 | 0.0635 | 0.0199 | 0.0227 | 0.1049 | 0.1933 | 0.0853 |
| Chronic kidney disease | 0.1280 | 0.0302 | 0.0041 | 0.1519 | 0.0723 | 0.0545 | 0.1005 | 0.0096 | 0.0543 | 0.1106 | ***0.2747*** | 0.0581 |
| Procedures | | | | | | | | | | | | |
| PCI | ***0.3073*** | 0.0591 | 0.0063 | ***0.3273*** | ***0.3134*** | 0.0288 | 0.1359 | 0.0375 | 0.0388 | 0.1773 | ***0.4081*** | 0.1022 |
| CABG | 0.0527 | 0.0556 | 0.0012 | 0.0160 | 0.2099 | 0.0084 | 0.0506 | 0.0170 | 0.0072 | 0.1367 | 0.0173 | 0.0384 |
| Prior and concurrent medications | | | | | | | | | | | | |
| Antiplatelets | 0.2456 | 0.0576 | 0.0613 | 0.2474 | 0.1452 | 0.0588 | 0.1176 | 0.0466 | 0.0591 | 0.2400 | ***0.3170*** | 0.1111 |
| Antipsychotics | 0.0990 | 0.0057 | 0.0802 | 0.0375 | 0.0480 | 0.0547 | 0.0309 | 0.0181 | 0.0140 | 0.0087 | 0.0898 | 0.0049 |
| Anticoagulants | 0.0188 | 0.0290 | 0.0039 | 0.1023 | 0.0499 | 0.0701 | 0.0581 | 0.0124 | 0.0014 | 0.0488 | 0.1233 | 0.0160 |
| Glucose-lowering medications | 0.0973 | 0.1182 | 0.0336 | 0.0175 | 0.0942 | 0.0374 | 0.0277 | 0.0260 | 0.0206 | 0.0072 | 0.0574 | 0.0570 |
| Loop and thiazide diuretics | 0.0498 | 0.0114 | 0.0204 | 0.1512 | 0.0187 | 0.0047 | 0.0990 | 0.0303 | 0.0057 | 0.0347 | 0.2109 | 0.0084 |
| Calcium channel blockers | 0.0339 | 0.0432 | 0.0586 | 0.0497 | 0.0385 | 0.1217 | 0.0215 | 0.0071 | 0.0166 | 0.0676 | 0.0554 | 0.0473 |
| Digoxin | 0.0459 | 0.0015 | 0.0444 | 0.0022 | 0.0428 | 0.0552 | 0.0509 | 0.0135 | 0.0197 | 0.0877 | 0.1232 | 0.0140 |
| Other lipid-lowering medications | 0.0681 | 0.0390 | 0.0575 | 0.0840 | 0.0576 | 0.0511 | 0.1085 | 0.0080 | 0.0056 | 0.0939 | 0.0791 | 0.0242 |
| Nitrates | 0.2126 | 0.0866 | 0.0322 | ***0.2781*** | 0.0788 | 0.0734 | 0.1301 | 0.0289 | 0.0202 | ***0.2527*** | ***0.2876*** | 0.1151 |
| Potassium-sparing diuretics | 0.1174 | 0.0113 | 0.0428 | 0.1212 | 0.0280 | 0.0334 | 0.0515 | 0.0581 | 0.0024 | 0.0412 | 0.0055 | 0.0700 |

**Abbreviations**: BB: beta-blockers, CABG: coronary artery bypass graft, COPD: chronic obstructive pulmonary disease, HFRS: Hospital Frailty Risk Score, PCI: percutaneous coronary intervention, RASI: renin-angiotensin system inhibitor

**Appendix 7:** Stratified analyses by age and sex in Australia

**Abbreviations**: BB: beta-blockers, CI: confidence interval RASI: renin-angiotensin system inhibitor

**Abbreviations**: BB: beta-blockers, CI: confidence interval RASI: renin-angiotensin system inhibitor

**Abbreviations**: BB: beta-blockers, CI: confidence interval RASI: renin-angiotensin system inhibitor

**References**

**1**.	WHOCC - ATC/DDD Index. https://www.whocc.no/atc_ddd_index/ (24 November 2022, date last accessed).

**2**.	Tolppanen A-M, Taipale H, Koponen M *et al.* Cohort profile: the Finnish Medication and Alzheimer’s disease (MEDALZ) study. BMJ Open 2016; 6: e012100.

**3**.	Healthcare Data Research | THIN Data. https://www.the-health-improvement-network.com (24 November 2022, date last accessed).

**4**.	Hsieh C-Y, Su C-C, Shao S-C *et al.* Taiwan’s National Health Insurance Research Database: past and future. Clin Epidemiol 2019; 11: 349–358.
